# Comprehensive analysis of SLC43A2 on the tumor immune microenvironment and prognosis of liver hepatocellular carcinoma

**DOI:** 10.3389/fgene.2022.911378

**Published:** 2022-09-16

**Authors:** Yan Liao, Junmei Weng, Lian Chen, Nan Hu, Xun Yuan, Jianhua Wang, Feng He, Yixin Cai, Qin Huang, Jianing Wang, Liu Huang

**Affiliations:** ^1^ Department of Oncology, Tongji Hospital, Tongji Medical College, Huazhong University of Science and Technology, Wuhan, Hubei, China; ^2^ The Reproductive Medical Center, The Seventh Affiliated Hospital of Sun Yat-sen University, Shenzhen, Guangdong, China; ^3^ Department of Immunology, School of Basic Medicine, Tongji Medical College, Huazhong University of Science and Technology, Wuhan, Hubei, China; ^4^ Department of Neurology and Laboratory of Clinical Genetics, Peking Union Medical College Hospital, Beijing, China; ^5^ Department of Thoracic Surgery, Tongji Hospital, Tongji Medical College, Huazhong University of Science and Technology, Wuhan, Hubei, China

**Keywords:** liver hepatocellular carcinoma (LIHC), SLC43A2, tumor immune microenvironment (TIME), prognostic risk score model, prognostic biomarker, therapeutic target

## Abstract

**Background:** Tumor cells outcompete T cells for methionine via overexpressing SLC43A2, causing T cells exhaustion. We explored the influence of SLC43A2 on tumor immune microenvironment (TIME), immune-related genes (IRGs) and the prognosis of liver hepatocellular carcinoma (LIHC) patients.

**Methods:** The TCGA-LIHC dataset (*n* = 374) and the ICGC-LIRI-JP-LIHC (*n* = 231) datasets were used as training and validation cohort, respectively. IRGs were obtained from ImmPort. Statistical analyses were performed using R (V 4.0.5). Online databases such as GEPIA, GSCALite, the Kaplan–Meier plotter, KEGG, TIMER2, and CMap were used for differential expression, immune infiltration, functional enrichment, survival, and drug-induced gene perturbation analysis.

**Results:** SLC43A2 expression was higher in LIHC, correlated with worse survival, but could not predict prognosis of LIHC separately (AUC = 0.467). SLC43A2 positively correlated with immune exhaustion markers (all *p* < 0.001) and with increased infiltration of Tregs, macrophages and myeloid-derived suppressor cells (MDSC) (all *p* < 0.05). SLC43A2 may regulate 120 IRGs. A prognostic risk score model was developed using the TCGA-LIHC cohort and validated by the ICGC-LIRI-JP cohort. Arachidonic acid, SB-202190 and guanethidine were identified as possible immunomodulators pharmacologically targeting SLC43A2 in LIHC.

**Conclusion:** SLC43A2 may create suppressive tumor microenvironment and regulate related IRGs, thus affecting the prognosis of LIHC. Arachidonic acid, SB-202190, and guanethidine may be worthy of further study as immunomodulators on SLC43A2.

## Introduction

Liver hepatocellular carcinoma (LIHC) is a highly prevalent and lethal cancer, and many therapeutics are being tested for this disease (Siegel et al., 2022). In recent years, immunotherapy has greatly improved the prognosis of patients with LIHC (Fu et al., 2019; Ruf et al., 2021). Immune cells depend on solute carrier transporters (SLCs) to transport metabolites involved in gene regulation and signal transduction (Chen and Chen, 2021). A previous study, published in *Nature*, indicated that tumor SLC43A2 (solute carrier family 43 member 2) could modify T cell methionine metabolism and lead to T cell depletion. Inhibiting tumor SLC43A2 can normalize methionine metabolism in effector T cells, rescue their function and improve anti-tumor immunity in preclinical models (Bian et al., 2020).

However, to the best of our knowledge, the relationships between SLC43A2 and tumor immune microenvironment (TIME) as well as prognosis in LIHC have not been reported. In addition to T cell exhaustion, whether SLC43A2 plays a role in modulating other immune cells infiltration or regulating immune-related genes (IRGs) in LIHC is unclear.

Using readily available cancer databases, we investigated into the predictive potential of SLC43A2 on LIHC prognosis and its relationship with tumor-infiltrating immune cells. Furthermore, we analyzed SLC43A2 related IRGs and constructed a prognostic risk score model to improve the accuracy of prognosis prediction in LIHC. Finally, we tried to find possible small molecule drugs (SMDs) which may combat the adverse effects of SLC43A2 in LIHC through the CMap database.

## Materials and methods

### Data collection and statistical analysis

Gene expression profiles and clinical information of 374 LIHC patients were downloaded and extracted from the TCGA databases as the TCGA-LIHC cohort (https://portal.gdc.cancer.gov/). In addition, RNA expression sequencing data and clinical information of 231 LIHC patients were obtained from the ICGC-LIRI-JP cohort (https://dcc.icgc.org/releases) for validation. The 1793 IRGs were obtained from Immunology Database and Analysis Portal database (ImmPort database, https://www.immport.org/shared/home).

The RNA-Seq gene expression data with workflow type of FPKM was transformed into TPM format and converted to log2 for further study. All statistical analyses were performed using R (https://www.r-project.org/, V 4.0.5). Corresponding R packages of *limma, survival, survminer, ROC*, *ClusterProfiler, Rms, DESeq2, Venn,* and *ggplot2* were used.

### Expression analysis of SLC43A2 between LIHC and normal tissues

GEPIA (http://gepia.cancer-pku.cn/) (Tang et al., 2019), GSCALite database (Liu et al., 2018), and R software were used for mRNA differential expression analysis of tumor/normal tissue. Information on SLC43A2 protein expression in normal liver tissue and LIHC tissue was extracted from the Human Protein Atlas (HPA, http://www.proteinatlas.org/) (Uhlen et al., 2017).

### Survival analyses of SLC43A2 in LIHC patients

GEPIA, the Kaplan–Meier (K–M) plotter database and R software were used to assess the effect of SLC43A2 expression on survival. Endpoints of survival including overall survival (OS), disease-specific survival (DSS), progression-free interval (PFI), and recurrence-free survival (RFS). Survival curves were generated by the Kaplan–Meier plots, and the results were displayed with hazard ratio (HR) and *p-*value.

### SLC43A2 expression and immune cell infiltration in LIHC

TIMER2 (https://cistrome.shinyapps.io/timer/) is a comprehensive resource for systematic analysis of immune infiltrates across diverse cancer types (Li et al., 2017). GSCALite (http://bioinfo.life.hust.edu.cn/web/GSCALite/) integrates cancer genomics analysis based on TCGA data in 33 cancers and normal tissue data from GTEx for gene set analysis in a one-in-all data analysis workflow (Liu et al., 2018). We used these two datasets to analyze the relationship between SLC43A2 expression, immune cell infiltration, and T cell exhaustion markers (Andrews et al., 2017; Masugi et al., 2017; Agresta et al., 2018; Pai et al., 2019; Wang et al., 2020; Wolf et al., 2020). Furthermore, a multivariate Cox proportional hazards regression model found immune cell subsets independently associated with survival adjusted for age, stage, and sex. A *p* value less than 0.05 was considered statistically significant.

### SLC43A2 related differentially expressed genes (DEGs) and functional enrichment analysis

According to the median levels of SLC43A2 expression, we divided LIHC patients into high/low expression groups. |log2 FC| >1.5 and *p* adjust <0.05 was used as the threshold value to screen for DEGs. In order to elucidate the functional profiles of the DEGs, Gene ontology (GO) (Walter et al., 2015) and Kyotoencyclopedia of genes and genomes (KEGG) (Kanehisa et al., 2017) analyses were used for functional enrichment analysis. The “*p*.adjust” function in the R programming language was used to adjust for multiple comparisons.

### Identification of IRGs that may be regulated by SLC43A2

Based on the 1793 IRGs and SLC43A2 related DEGs, we obtained 120 IRGs that may be regulated by SLC43A2, of which 22 OS related IRGs were found by Univariate Cox regression analysis. Furthermore, Least Absolute Shrinkage and Selection Operator (LASSO) Cox regression model was used for signature construction (Tibshirani, 1997). Ultimately, 5 genes (*LECT2, CXCL8, FABP6, NR0B1, PGLYRP4*) were selected from the 22 OS associated SLC43A2 related IRGs to construct a prognostic prediction model.

### Identification and validation of the prognostic risk score model

Based on the expression levels of 5 genes and corresponding regression coefficients, the risk score of patients in TCGA-LIHC cohort were calculated. Risk score = sum (corresponding coefficient × each gene’s expression). TCGA-LIHC patients were divided into high-risk and low-risk groups by the median of risk score. Principal component analysis (PCA) was used for dimensionality analysis. The survival analysis was visualized using K-M survival curves with log-rank testing. The ICGC-LIRI-JP cohort was used to validate the prognostic value of the risk score model. Multivariate cox regression analysis and Receiver operating curves (ROC) were used to estimate whether risk score in combination with stage had better prognostication. In addition, a nomogram was constructed and assessed by the calibration curves to predict 1-, 3-, and 5-year OS rate. When the curve approaches to the 45-degree line, it represents the best prognostic prediction.

### Identification of SMDs for reversing immunosuppressive of SLC43A2 in LIHC

The Connectivity Map (CMap) v2.0 (https://portals.broadinstitute.org/cmap) (Lamb et al., 2006) was used to identify the SMDs that may reverse the immunosuppressiveness of SLC43A2. CMap provide transcriptomic data for drug treatments. We identified SMDs possessing the lowest risk score of the 5 genes involved in the risk score model (the connectivity enrichment value was *>* 0.8, *p <* 0.01). And the 3D conformers of the top 3 candidate therapeutic agents were downloaded (https://go.drugbank.com *,* https://www.ncbi.nlm.nih.gov/geoprofile).

## Results

### Associations between SLC43A2 expression and clinicopathologic factors in LIHC

**FIGURE 1:**
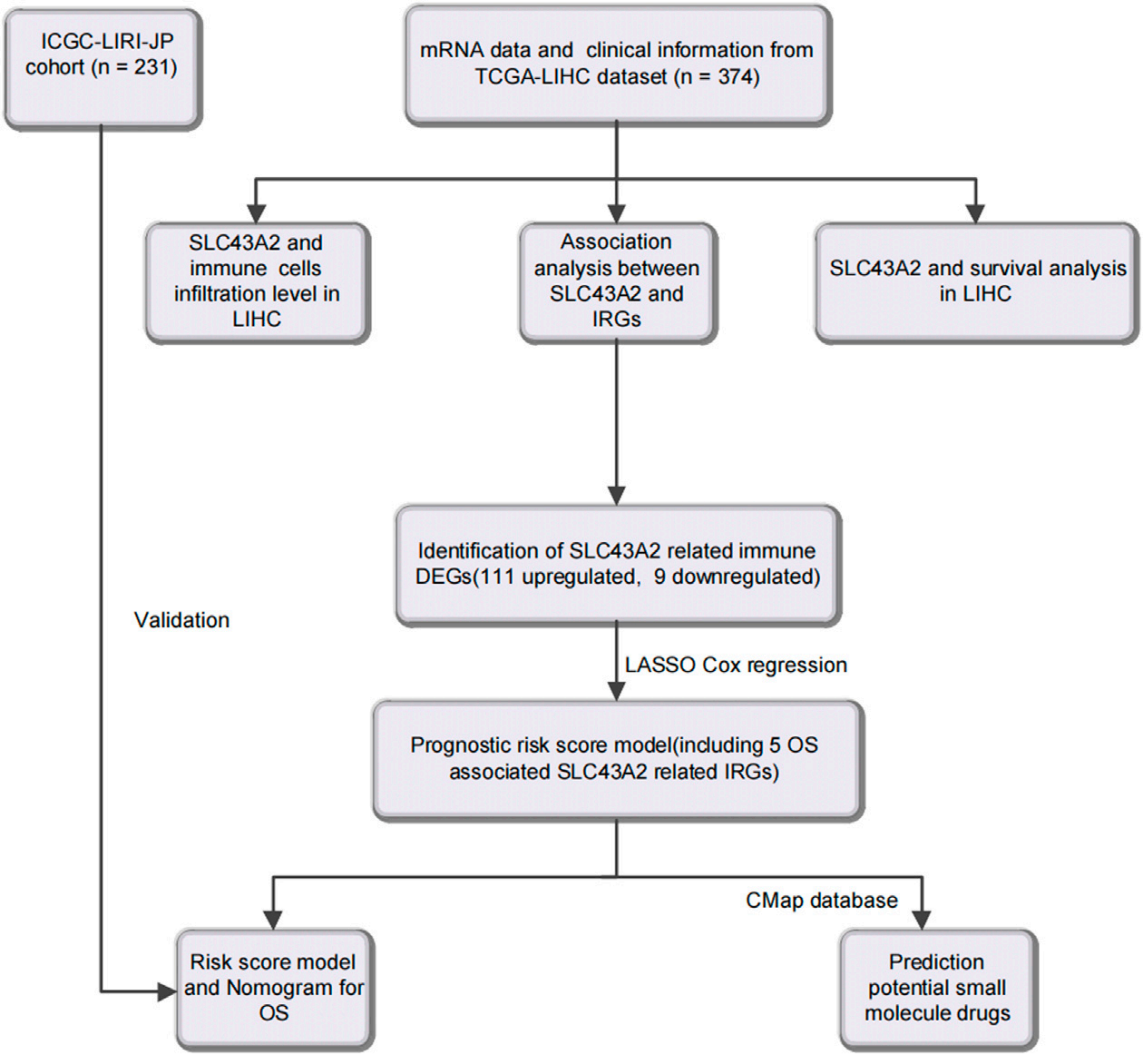
Flow diagram of this study.

**FIGURE 2:**
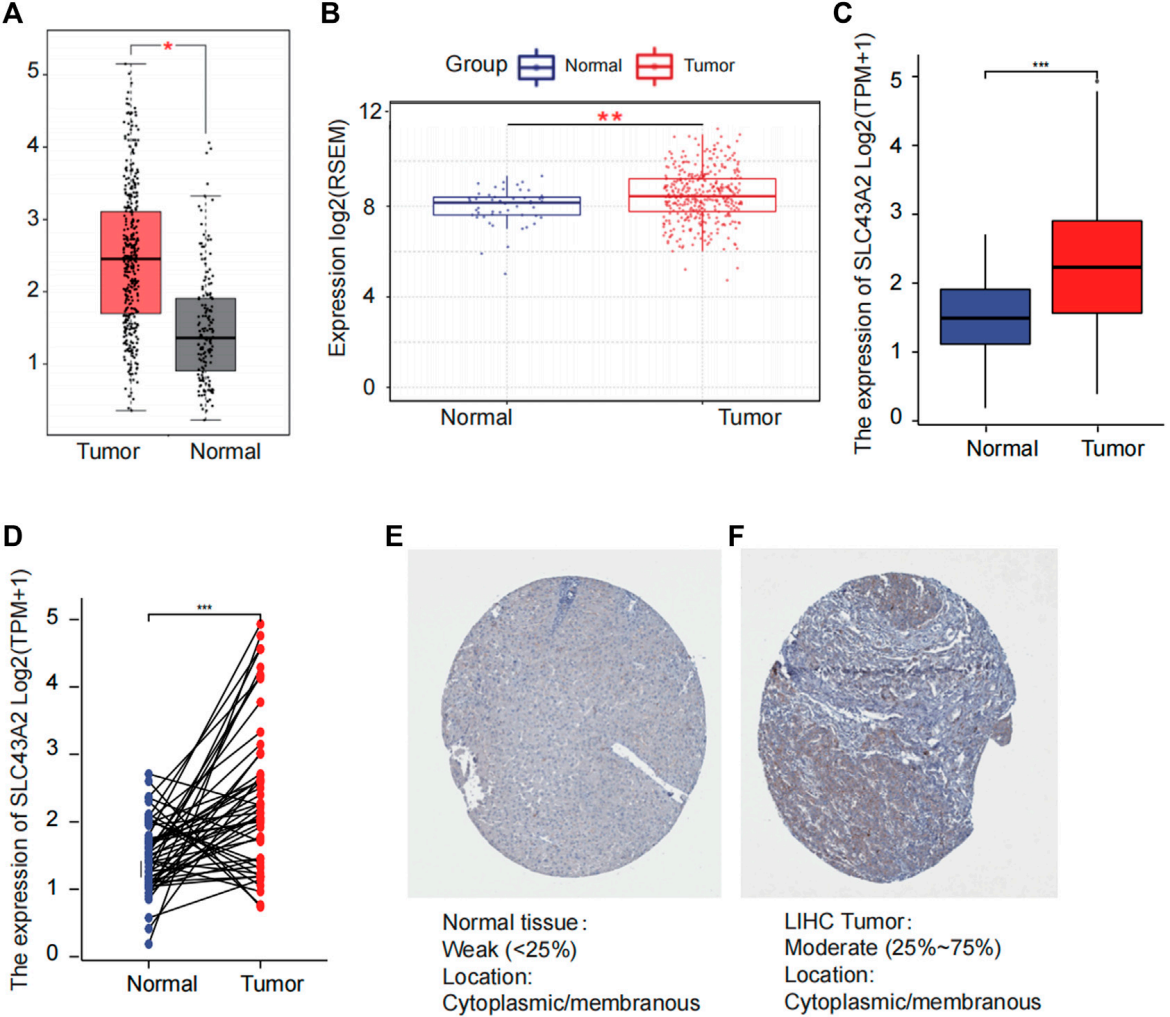
Difference of SLC43A2 expression between LIHC and normal tissues. The difference of the mRNA expression of SLC43A2 between LIHC and normal tissues in GEPIA **(A)** and GSCALite **(B)** datasets. The mRNA expression levels of SLC43A2 in 374 LIHC samples and 50 normal samples **(C)**. The mRNA expression levels of SLC43A2 in 50 LIHC and matched-adjacent normal samples **(D)**. The difference of protein levels of SLC43A2 between LIHC and normal tissues based on HPA **(E, F)**. (ns, no significance, *p* ≥ 0.05; *p < 0.05; **p < 0.01; ***p < 0.001).

Figure 1 was the workflow of our research. Compared to normal tissue, LIHC had considerably higher expression level of SLC43A2. Figure 2 showed the expression differences of SLC43A2 in GEPIA (Figure 2A, *p* < 0.05), GSCA (Figure 2B, *p* < 0.001), TCGA-LIHC unpaired (Figure 2C, *p* < 0.001) and paired (Figure 2D, *p* < 0.001) analyses respectively. Besides, immunohistochemical staining from HPA indicated the upregulation of SLC43A2 protein in LIHC [Figures 2E,F, Normal tissue: Weak (<25%), LIHC Tumor: Moderate (25%–75%)]. High expression of SLC43A2 had higher levels of AFP (25 vs. 4 ng/ml, *p =* 0.012), while T stage, N stage, M stage, age, Albumin (g/dl), or Body Mass Index (BMI) had no significant differences (Table 1).

**TABLE 1:** The association between SLC43A2 expression and clinicopathological variables.

Characteristic	Low expression of SLC43A2	High expression of SLC43A2	*p* Value
n	187	187	
T stage, n (%)			0.791
T1	94 (25.3%)	89 (24%)	
T2	43 (11.6%)	52 (14%)	
T3	40 (10.8%)	40 (10.8%)	
T4	7 (1.9%)	6 (1.6%)	
N stage, n (%)			0.361
N0	131 (50.8%)	123 (47.7%)	
N1	1 (0.4%)	3 (1.2%)	
M stage, n (%)			0.122
M0	134 (49.3%)	134 (49.3%)	
M1	4 (1.5%)	0 (0%)	
Age, median (IQR)	62 (52, 69)	61 (51, 68)	0.477
AFP (ng/ml), median (IQR)	9 (4, 114)	25 (5, 535.5)	0.012*
Albumin (g/dl), median (IQR)	4 (3.5, 4.3)	4 (3.5, 4.3)	0.842
BMI, median (IQR)	25.01 (22.01, 28.66)	24.16 (21.32, 28.66)	0.594

IQR, interquartile range; AFP, Alpha-Feto Protein; BMI, Body mass index.

### Associations between SLC43A2 expression and survival in LIHC

**FIGURE 3:**
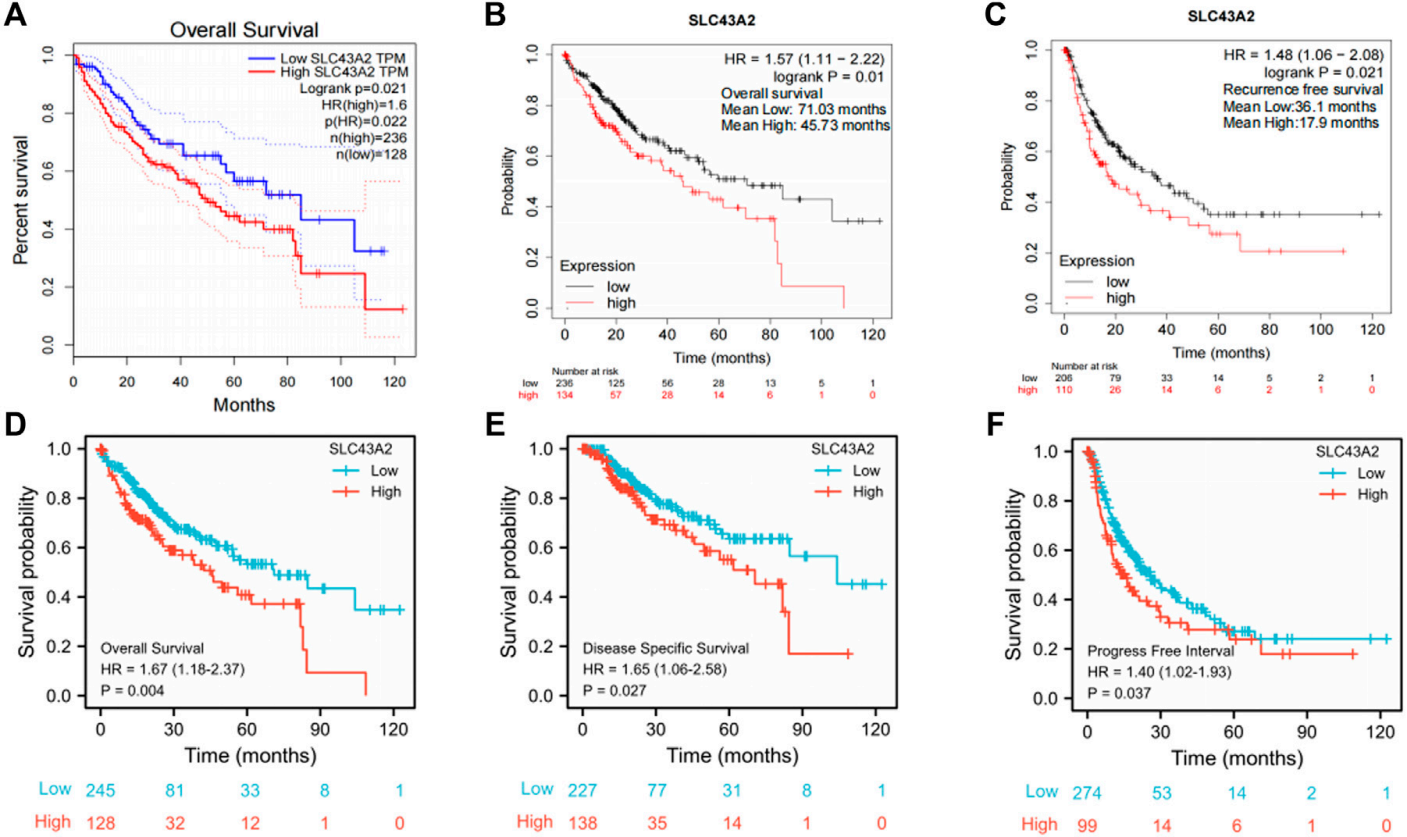
Correlations between SLC43A2 expression and survival in LIHC. OS curves in LIHC patients with SLC43A2-high or -low expression by using GEPIA [**(A)**, logrank *p* = 0.021, HR = 1.6]. OS **(B)** and RFS **(C)** in LIHC patients with SLC43A2-high or -low expression by using the Kaplan Meier plotter database (logrank *p* = 0.01 and logrank *p* = 0.021, respectively). OS, DSS, and PFI between LIHC patients with high or low SLC43A2 expression in TCGA-LIHC cohort [**(D–F)**, *p* = 0.004, 0.027, and 0.037 respectively]. OS, overall survival; DSS, disease-specific survival; PFI, progression-free interval; RFS, recurrence-free survival.

Elevated SLC43A2 expression had a negative effect on OS of LIHC (GEPIA database analysis, HR = 1.6, log-rank *p* = 0.021) (Figure 3A). The K-M plotter database also showed that high-levels of SLC43A2 were associated with worse OS and RFS (45.73 vs. 71.03 months, log-rank *p =*0.01; 17.9 vs. 36.1 months, log-rank *p =* 0.021 respectively; Figures 3B,C). Moreover, the 10-year OS, DSS, and PFI were significantly lower in patients with higher SLC43A2 expression (in TCGA-LIHC cohort, HR = 1.67, 95%CI = 1.18–2.37, *p =* 0.004; HR = 1.65, 95%CI = 1.06–2.58, *p* = 0.027; HR = 1.40, 95%CI = 1.02–1.93, *p* = 0.037; respectively, Figures 3D–F).

### Associations between SLC43A2 expression and immune infiltration in LIHC

Multivariate Cox proportional hazards regression model showed that CD8^+^ T cells, endothelial cells, and hematopoietic stem cells could independently predict longer OS for LIHC patients. While Th2 cell, T cell regulatory (Tregs), macrophage, myeloid dendritic cell (MDC), and myeloid-derived suppressor cells (MDSC) predicted worse OS (Table 2, all *p* < 0.05, adjusted by age, stage, and sex).

**TABLE 2:** The associations analyzing by Multivariate Cox proportional hazards regression models between immune infiltrates and overall survival of LIHC in TCGA (*n* = 371).

	coef	HR	95% CI_low	95% CI_upper	*p* Value
T cell CD8^+^	−2.755	0.064	0.008	0.526	0.011**
T cell CD4^+^ Th2	5.401	221.664	18.139	2708.793	0.000**
T cell regulatory (Tregs)	5.819	336.527	1.376	82278.749	0.038**
B cell	−0.923	0.397	0.047	3.391	0.399
Neutrophil	0.874	2.397	0.615	9.334	0.208
Macrophage	2.093	8.113	2.287	28.773	0.001**
Myeloid dendritic cell	13.301	5.978e + 13	19.217	1.859e + 10	0.012*
NK cell activated	−2.646	0.071	0.001	10.063	0.295
Mast cell activated	−2.839	0.059	0.003	1.042	0.053
Cancer associated fibroblast	0.873	2.393	0.004	1529.490	0.791
Common lymphoid progenitor	15.179	3.910e + 6	0.630	2.427e + 13	0.057
Common myeloid progenitor	−42.329	0.000	0.000	771.004	0.090
Endothelial cell	−4.782	0.008	0.000	0.817	0.041*
Eosinophil	92.144	1.04e + 40	0.000	6.293e + 87	0.101
Granulocyte-monocyte progenitor	−11.595	0.000	0.000	111.900	0.164
Hematopoietic stem cell	−2.690	0.068	0.009	0.528	0.010*
T cell follicular helper	1.505	4.505	0.025	806.634	0.570
T cell gamma delta	2.445	11.535	0.003	49994.354	0.567
T cell NK_XCELL	−2.269	0.103	0.000	419.792	0.593
MDSC	5.705	300.361	18.296	4930.851	0.000***

^#^, Adjusted by Age, Stage and Sex. *p＜0.05; **p＜0.01 ***p＜0.001. MDSC, myeloid derived suppressor cell.

**FIGURE 4:**
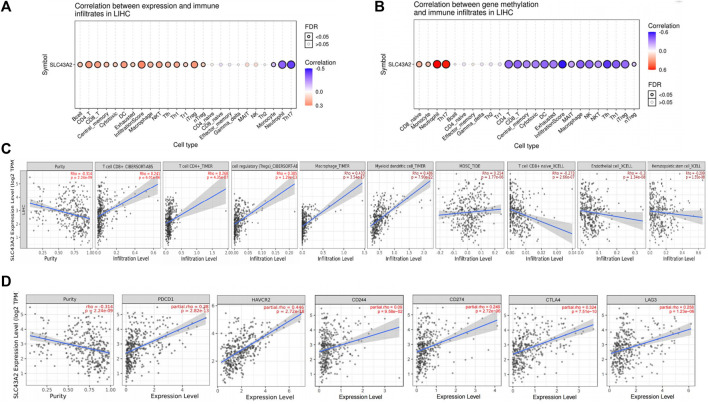
Correlation of SLC43A2 expression with immune infiltration level in LIHC. Correlations between SLC43A2 expression **(A)**, SLC43A2 methylation **(B)** and the relative abundances of 24 immune cells by using GSCA dataset. Bubble size are correlate with FDR significance. Black outline border indicates FDR ≤ 0.05, FDR: the false discovery rate. **(A, B)**. Correlation of SLC43A2 expression with immune infiltration level in LIHC using TIMER2 dataset **(C)**. Correlation of SLC43A2 expression with the abundance of PDCD1, TIM3(HAVCR2), CD244, CD274, CTLA4, and LAG3 in LIHC using TIMER2 dataset **(D)**.

We found that SLC43A2 was positively associated with Tregs, macrophage and MDSC infiltration in LIHC in both the GSCA dataset (Figures 4A,B) and the TIMER2 dataset (Figure 4C). SLC43A2 was also positively correlated with the expression of T cell exhaustion markers PDCD1, TIM3(HAVCR2), CD244, CD274, CTLA4, and LAG3 in LIHC (*p <* 0.001) (Figure 4D).

### SLC43A2 related DEGs and functional enrichment analysis

**FIGURE 5:**
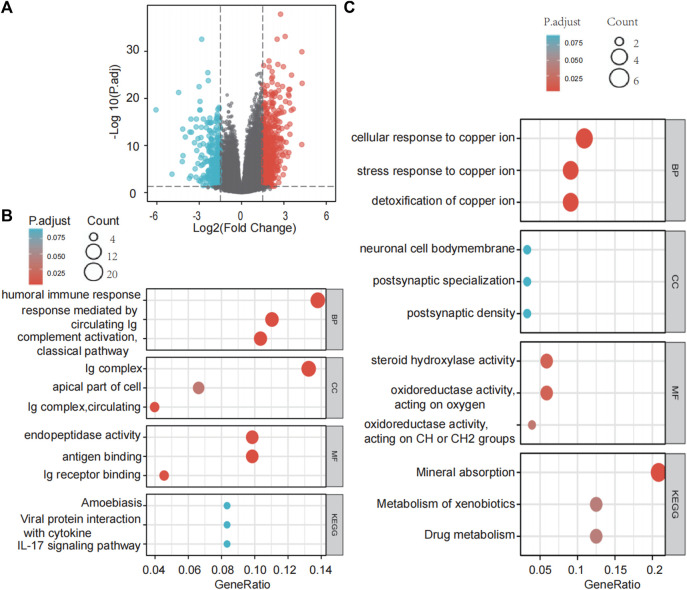
Differential expression analysis of SLC43A2 and the functional enrichment analysis of these DEGs. Volcano plots of the DEGs (|log2(FC)|>1.5 & *p*. adjust <0.05) **(A)**. GO enrichment and KEGG Pathway analyses of 638 genes upregulated and 284 genes downregulated in SLC43A2 **(B** and **C)**. BP, biological process; CC, cellular component; MF, molecular function.

Differential gene expression analysis in LIHC patients with high or low SLC43A2 expression identified 638 upregulated genes and 284 downregulated genes according to the standard of *p*.adjust <0.05 and |log2 FC| > 1.5 (Figure 5A). GO enrichment and KEGG pathway analysis revealed that genes upregulated by SLC43A2 were enriched in several immune-related pathways such as humoral immune response, circulating immunoglobulin(lg), antigen binding (Figure 5B, *p*.adjust <0.05). Genes downregulated by SLC43A2 were enriched in pathways associated with copper ion (Figure 5C, *p*.adjust <0.001).

### Associations between SLC43A2 and IRGs in LIHC

**FIGURE 6:**
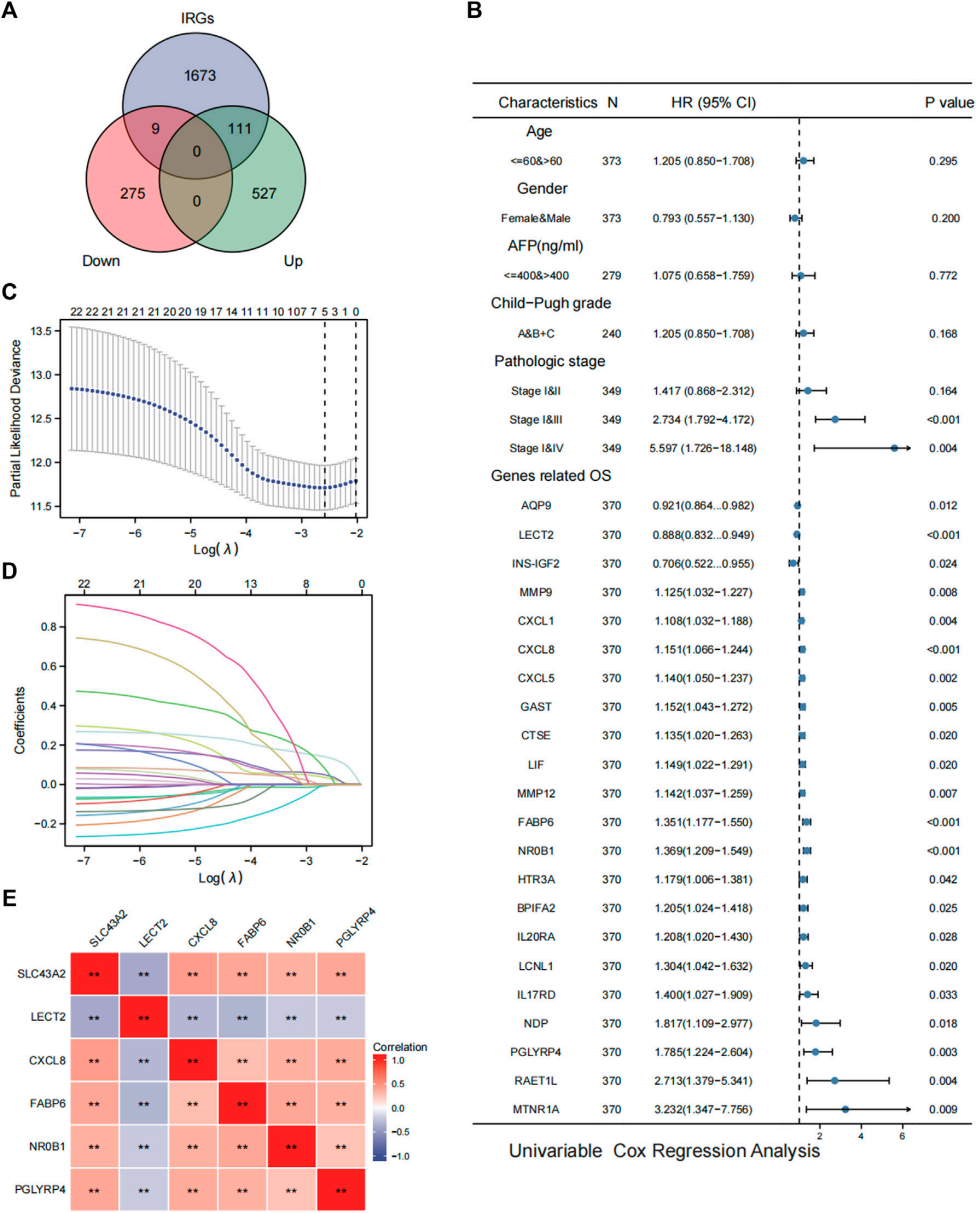
Analysis of SLC43A2 related IRGs in LIHC. Venn diagrams showing the intersection of the IRGs and SLC43A2-related DEGs in LIHC, which was defined as SLC43A2-related IRGs **(A)**. Forest plots showing the results of univariate Cox regression analysis of OS based on clinicopathological factors (such as age, gender, AFP, Child-Pugh grade and stage) and SLC43A2 IRGs [**(B)**, only the 22 IRGs significantly associated with OS were shown]. Five genes (*LECT2*, *CXCL8*, *FABP6*, *NR0B1*, *PGLYRP4*) were furtherly selected from the 22 SLC43A2 related IRGs by the LASSO Cox regression **(C, D)**. Correlation of SLC43A2 with the 5 genes (LECT2, CXCL8, FABP6, NR0B1, and PGLYRP4) (**p < 0.01) **(E)**.

SLC43A2 related IRGs were identified with the intersection of the IRGs and SLC43A2-related DEGs in LIHC. As shown in Figure 6A, we identified 111 upregulated and 9 downregulated SLC43A2 related IRGs. Of the 120 SLC43A2 related IRGs, 22 IRGs were found to be associated with OS and unassociated with age, gender, AFP (ng/ml), and Child–Pugh grade (Figure 6B). To minimize overfitting, LASSO Cox regression was used to select 5 genes (*CXCL8*, *FABP6*, *NR0B1*, *PGLYRP4* and *LECT2*) (Figures 6C,D), all of which were closely related to SLC43A2 (Figure 6E) (*p <* 0.01).

### Development and external validation of the prognostic risk score model

**FIGURE 7:**
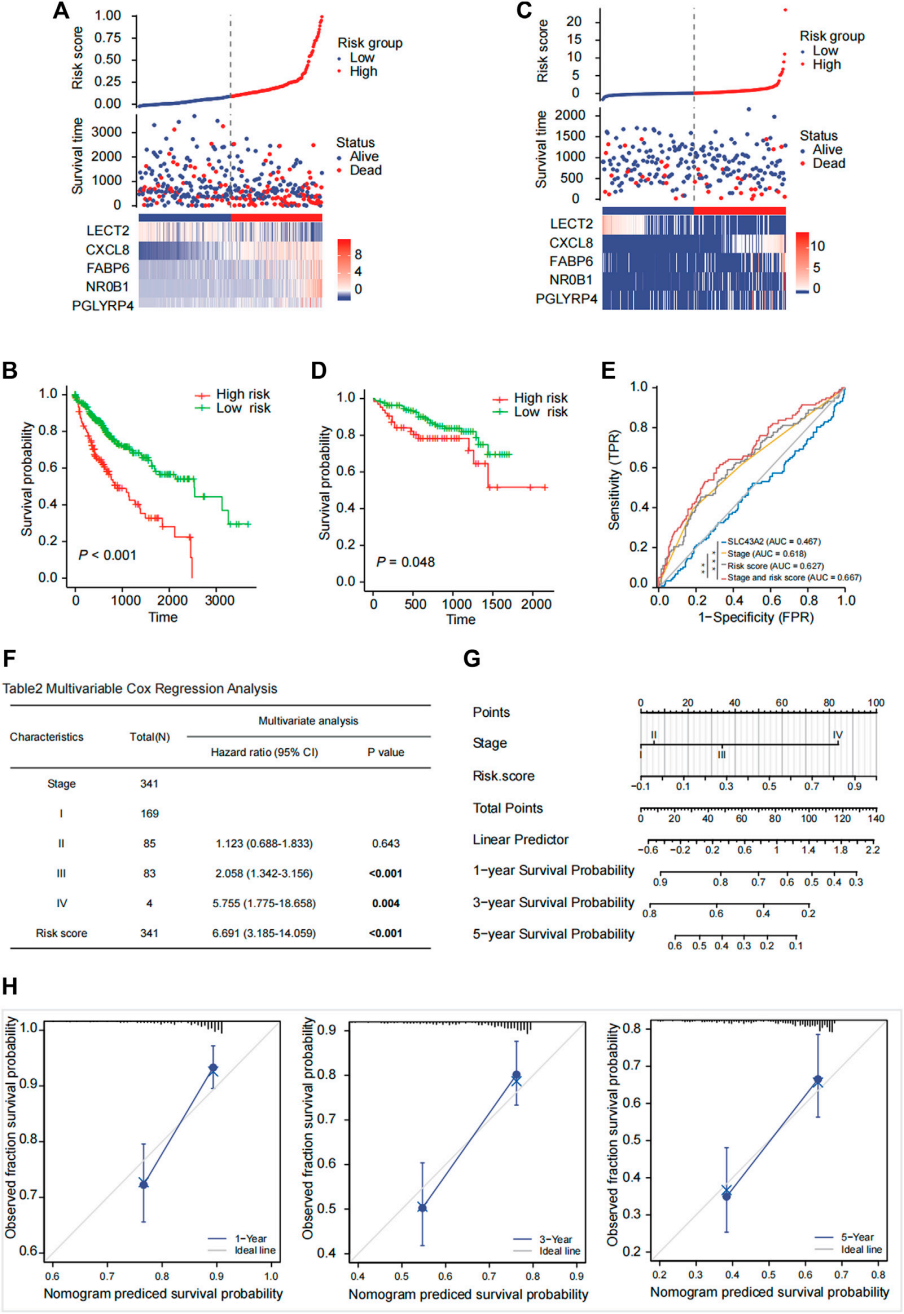
Risk score model prediction and validation. Building a five-gene signature risk score in the TCGA-LIHC cohort **(A)** with the OS probability for high and low risk patients **(B)** and validating in the ICGC-LIRI-JP cohort **(C, D)**. The risk score was calculated based on the expression of 5 genes (*LECT2*, *CXCL8*, *FABP6*, *NR0B1*, *PGLYRP4*). The Kaplan-Meier survival curves of OS between high-risk and low-risk groups were shown in TCGA-LIHC cohort [**(B)**, *p* < 0.001] and in the ICGC-LIRI-JP cohort [**(D)**, *p* = 0.048]. ROC analysis to compare SLC43A2, stage, risk score, stage and risk score in predicting survival [**(E)**, AUC = 0.467, 0.618, 0.627, 0.667]. Multivariate Cox regression analysis of OS based on stage and risk score in the TCGA cohort **(F)**. A nomogram was constructed based on stage and risk score **(G)**. Calibration plot evaluating the predictive accuracy of the nomogram at 1-, 3-, and 5-year survival **(H)**.

The risk score of each patient was calculated based on gene expression and corresponding regression coefficients. The gray dashed line in Figures 7A,C represents the cutoff value point and divided the cohort into two groups with the left part represents low‐risk score group and the right part represents high‐risk score group. Point plot shows high‐ and low‐risk score patients groups divided by the median cutoff values and represented by color: Blue represents low‐risk score group, and red represents high‐risk score group. The scatter plot of ordered risk scores shows OS status of each patient. Heatmap shows the expression profile of the 5-gene signature. Each column indicates a patient in the low-risk score group (blue) and high-risk score group (red). Each row represents the level of gene expression associated with survival (red represents high, and blue represents low). The TCGA-LIHC cohort was divided into high-risk and low-risk groups according to the median risk score (Figure 7A). The OS difference between these two groups was significant (Figure 7B, *p* < 0.001). Results were similar in the validation cohort of ICGC-LIRI-JP (Figures 7C,D, *p* = 0.048). The clustered heat maps showed that the expression of prognostic genes *CXCL8, FABP6, NR0B1,* and *PGLYRP4* was upregulated in the high-risk group, while the expression of *LECT2* was downregulated in the high-risk group (Figures 7A,C).

### Construction of a nomogram based on the prognostic risk score and stage

Pathological stage (stage III: HR:2.058; 95% CI:1.342–3.156; stage IV: HR: 5.755; 95% CI: 1.775–18.658) and high-risk score (HR: 6.691; 95% CI: 3.185–14.059) were independent risk factors for OS in the Multivariate Cox regression analysis (Figure 7F). The ROC curve analysis showed acceptable discrimination with AUCs of 0.467, 0.618, 0.627, and 0.667 at SLC43A2, stage, risk score, and stage and risk score, respectively. The diagnostic efficiency of stage and risk score was better than that of stage (Figure 7E, ∗*p* < 0.05). We then developed a nomogram to predict 1-, 3-, and 5-year overall survival based on the risk score and pathologic stage in LIHC (Figure 7G). Moreover, the calibration plot (Figure 7H) demonstrated optimal predictive accuracy with predicted survival rate highly consistent with actual survival.

### Identification of SMDs for reversing immunosuppressive of SLC43A2 in LIHC 

We furtherly analyzed the CMap database to predict potential SMDs for reversing immunosuppressive of SLC43A2 in LIHC. The top three SMDs were revealed using the highest absolute enrichment values and *p <* 0.01 (Table 3). The 3D conformers for the top three most significant candidates are shown in Supplementary Figure S1.

**TABLE 3:** Top 3 small molecules with the highest absolute enrichment values identified with risk score model DEGs.

Rank	Cmap name	Enrichment	*p* < 0.01	Description
1	arachidonic acid	−0.925	0.00072	An unsaturated, essential fatty acid; A precursor in the biosynthesis of prostaglandins, thromboxanes, and leukotrienes
2	SB-202190	−0.801	0.0007	p38 MAPK inhibitor
5	guanethidine	−0.877	0.00377	Inhibiting or interfering with the release distribution of norepinephrine

## Discussion

LIHC is one of the most frequently occurring cancers worldwide, ranked 3rd in global incidence by the International Agency for Research on Cancer (WHO, 2022). However, the clinical response of some LIHC patients to this treatment has been unsatisfactory (Fu et al., 2019). Previous studies have shown that CD8^+^ T cells were critical to the efficacy of immunotherapy (Fan and Rudensky, 2016; Guo et al., 2020). SLC43A2 was found to impair T cell function, partly because the tumor cells highly expressed SLC43A2 and then outcompeted T cells for methionine (Bian et al., 2020). However, the effects of SLC43A2 on TIME, IRGs, and prognosis of LIHC have not been reported.

We firstly reported that high SLC43A2 expression was associated with worse OS and strong enrichment of inhibitory immune cells such as Tregs, macrophages, MDC, and MDSC in LIHC. Consistent with previous reports (Ma et al., 2016; Zhang et al., 2019a; Fu et al., 2019; Xu et al., 2022) we found that Tregs, macrophages, MDC and MDSC predicted worse OS, even in Multivariate Cox proportional hazards regression analysis (Table 2, all *p* < 0.05, adjusted by age, stage and sex). This may partly explain why patients with high SLC43A2 have lower survivorship.

Intriguingly, we found that SLC43A2 was associated with higher CD8^+^ T cell, higher T cell exhaustion markers, and lower levels of naive CD8^+^ T cell. Genes that may be influenced by SLC43A2 were enriched in immune pathways. These results support the conclusion that SLC43A2 could lead to CD8^+^ T cell exhaustion and may affect TIME.

Our study was the first to report on the relationship between SLC43A2 and IRGs. We found 120 IRGs that may be influenced by SLC43A2 and then identified 5 IRGs (*CXCL8*, *FABP6*, *NR0B1*, *PGLYRP4* and *LECT2*) to establish a risk score model to predict the OS of LIHC. *CXCL8, FABP6,* and *NR0B1* promote tumor growth in LIHC (Bar-Peled et al., 2017; Zhang et al., 2019b; Sun et al., 2019; Huang et al., 2015). *PGLYRP4* plays a role in inflammation and immune cell recruitment (Dabrowski et al., 2019; Karami et al., 2021). *LECT2* inhibits the tumorigenicity of the LIHC cells *in vivo* (Zhu et al., 2022; Ong et al., 2011). This risk score model was developed using the TCGA-LIHC cohort and validated using the ICGC-LIRI-JP cohort. Combining risk score and pathologic stage was the most effective method for predicting OS of LIHC patients.

SLC43A2 played an important role in suppressing anti-tumor immunity, however, precise inhibition of SLC43A2 of tumor cells *in vivo* was difficult since it was widely expressed in various tissues such as the placenta, small intestine enterocytes, kidney epithelium, and peripheral blood leukocytes (Chen and Chen, 2022). Thus we tried to find SMDs that may reverse the immunosuppressive role of SLC43A2. Based on the CMap database (Lamb et al. 2006), which collected expression data from cells following exposure to drugs and other perturbations, we found that our three drugs—arachidonic acid, SB-202190, and guanethidine would lead to lower expression of *CXCL8, FABP6, NR0B1, and PGLYRP4* and higher expression of *LECT2* in LIHC. In our risk score model, *CXCL8*, *FABP6*, *NR0B1*, and *PGLYRP4* were recognized as risk genes while *LECT2* was a protective gene. This is to say, after being treated by the 3 SMDs, the risk score of LIHC could decrease. Arachidonic acid, a phospholipase A2 metabolite, reduced cell and migration and increased apoptosis of breast cancer and lung cancer (Muzio et al., 2006). SB-202190, an ATP competitive antagonist of the p38 stress-activated protein kinases (Shanware et al., 2009), could be valid for inhibiting tumor cell migration, invasion, and metastasis in LIHC (Yang et al., 2010; Düzgün Ş et al., 2017). Guanethidine could inhibit the release of noradrenaline, which usually served as an immunosuppressor to improve a suitable environment for tumor cells to grow and metastasize (Sarkar et al., 2013)]. To the best of our knowledge, these three drugs have not been adequately studied in LIHC.

Our study is based on bioinformatic analysis and lacks experimental verification, but we have several suggestions for further research on SLC43A2. First, the detailed mechanism of the impact of SLC43A2 on immune infiltration in LIHC needs to be verified *in vitro* and *in vivo*. Second, how SLC43A2 alters the expression of the 5 IRGs in LIHC needs to be verified. Third, verify whether the selected SMDs (arachidonic acid, SB-202190 and guanethidine), could affect anti-tumor immunity and achieve therapeutic effects.

In conclusion, the high expression of SLC43A2 was significantly associated with the poor survival and T cell exhaustion in LIHC. SLC43A2 may influence IRGs expression and lead to suppressive TIME. Our risk score model could improve the predictive efficiency of SLC43A2 and the pathologic TNM stage on OS. Arachidonic acid, SB-202190, and guanethidine may reverse the adverse role of SLC43A2 in LIHC.

## Data Availability

The datasets presented in this study can be found in online repositories. The names of the repository/repositories and accession numbers can be found in the article/Supplementary Material.
