# Supplementary figures and images for: Comprehensive analysis of SLC43A2 on the tumor immune microenvironment and prognosis of liver hepatocellular carcinoma
Source: Front Genet. 2022 Sep 16;13:911378. doi: 10.3389/fgene.2022.911378 (PMC9523210; doi:10.3389/fgene.2022.911378)

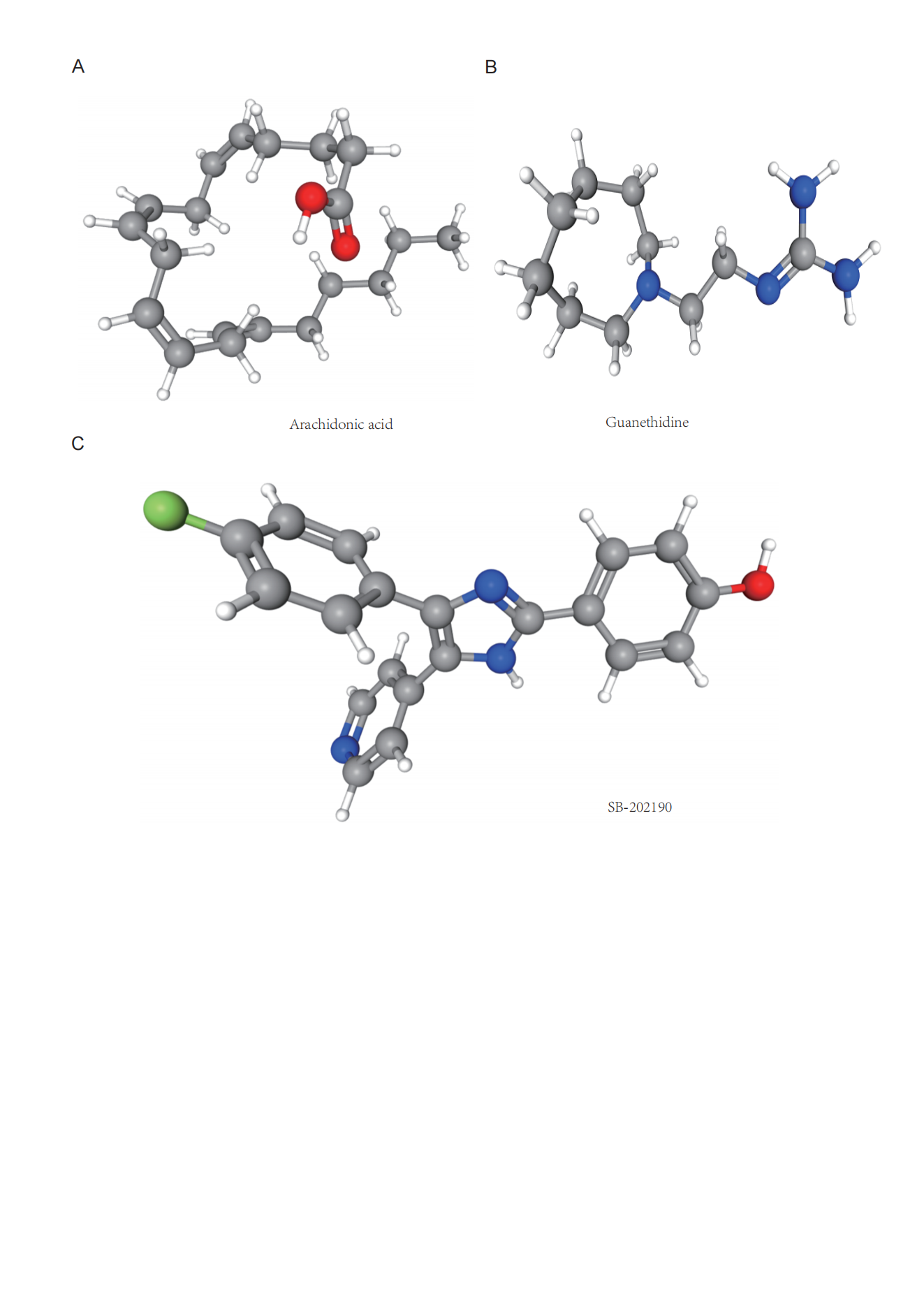

Supplement: Supplementary file 2 [file Image1.tif]
